# Global Assessment of Dengue Virus-Specific CD4^+^ T Cell Responses in Dengue-Endemic Areas

**DOI:** 10.3389/fimmu.2017.01309

**Published:** 2017-10-13

**Authors:** Alba Grifoni, Michael A. Angelo, Benjamin Lopez, Patrick H. O’Rourke, John Sidney, Cristhiam Cerpas, Angel Balmaseda, Cassia G. T. Silveira, Alvino Maestri, Priscilla R. Costa, Anna P. Durbin, Sean A. Diehl, Elizabeth Phillips, Simon Mallal, Aruna D. De Silva, Godwin Nchinda, Celine Nkenfou, Matthew H. Collins, Aravinda M. de Silva, Mei Qiu Lim, Paul A. Macary, Filippo Tatullo, Tom Solomon, Vijaya Satchidanandam, Anita Desai, Vasanthapram Ravi, Josefina Coloma, Lance Turtle, Laura Rivino, Esper G. Kallas, Bjoern Peters, Eva Harris, Alessandro Sette, Daniela Weiskopf

**Affiliations:** ^1^Division of Vaccine Discovery, La Jolla Institute for Allergy and Immunology, La Jolla, CA, United States; ^2^Laboratorio Nacional de Virología, Centro Nacional de Diagnóstico y Referencia, Ministerio de Salud, Managua, Nicaragua; ^3^Division of Clinical Immunology and Allergy, School of Medicine, University of São Paulo, São Paulo, Brazil; ^4^Johns Hopkins University Bloomberg School of Public Health, Baltimore, MD, United States; ^5^Vaccine Testing Center, Department of Medicine, Larner College of Medicine, University of Vermont, Burlington, VT, United States; ^6^Institute for Immunology and Infectious Diseases, Murdoch University, Perth, WA, Australia; ^7^Department of Medicine, Vanderbilt University Medical Center, Nashville, TN, United States; ^8^Genetech Research Institute, Colombo, Sri Lanka; ^9^Chantal BIYA International Reference Centre for Research on the Prevention and Management of HIV/AIDS CIRCB, Yaoundé, Cameroon; ^10^Department of Microbiology and Immunology, University of North Carolina School of Medicine, Chapel Hill, NC, United States; ^11^Emerging Infectious Disease Programme, Duke-NUS Medical School, Singapore, Singapore; ^12^Immunology Programme, Department of Microbiology and Immunology, Life Sciences Institute, National University of Singapore, Singapore, Singapore; ^13^Institute of Infection and Global Health, University of Liverpool, Liverpool, United Kingdom; ^14^National Institute for Health Research, Health Protection Research Unit in Emerging and Zoonotic Infections, University of Liverpool, Liverpool, United Kingdom; ^15^Department of Microbiology and Cell Biology, Indian Institute of Science, Bangalore, India; ^16^Neurovirology, National Institute of Mental Health and Neurosciences (NIMHANS), Bangalore, India; ^17^Division of Infectious Diseases and Vaccinology, School of Public Health, University of California, Berkeley, Berkeley, CA, United States

**Keywords:** dengue virus, CD4^+^ T cells, HLA, epitope, adaptive immunity

## Abstract

**Background:**

Dengue is a major public health problem worldwide. Assessment of adaptive immunity is important to understanding immunopathology and to define correlates of protection against dengue virus (DENV). To enable global assessment of CD4^+^ T cell responses, we mapped HLA-DRB1-restricted DENV-specific CD4^+^ T cell epitopes in individuals previously exposed to DENV in the general population of the dengue-endemic region of Managua, Nicaragua.

**Methods:**

HLA class II epitopes in the population of Managua were identified by an *in vitro* IFNγ ELISPOT assay. CD4^+^ T cells purified by magnetic bead negative selection were stimulated with HLA-matched epitope pools in the presence of autologous antigen-presenting cells, followed by pool deconvolution to identify specific epitopes. The epitopes identified in this study were combined with those previously identified in the DENV endemic region of Sri Lanka, to generate a “megapool” (MP) consisting of 180 peptides specifically designed to achieve balanced HLA and DENV serotype coverage. The DENV CD4MP_180_ was validated by intracellular cytokine staining assays.

**Results:**

We detected responses directed against a total of 431 epitopes, representing all 4 DENV serotypes, restricted by 15 different HLA-DRB1 alleles. The responses were associated with a similar pattern of protein immunodominance, overall higher magnitude of responses, as compared to what was observed previously in the Sri Lanka region. Based on these epitope mapping studies, we designed a DENV CD4 MP_180_ with higher and more consistent coverage, which allowed the detection of CD4^+^ T cell DENV responses *ex vivo* in various cohorts of DENV exposed donors worldwide, including donors from Nicaragua, Brazil, Singapore, Sri Lanka, and U.S. domestic flavivirus-naïve subjects immunized with Tetravalent Dengue Live-Attenuated Vaccine (TV005). This broad reactivity reflects that the 21 HLA-DRB1 alleles analyzed in this and previous studies account for more than 80% of alleles present with a phenotypic frequency ≥5% worldwide, corresponding to 92% phenotypic coverage of the general population (i.e., 92% of individuals express at least one of these alleles).

**Conclusion:**

The DENV CD4 MP_180_ can be utilized to measure *ex vivo* responses to DENV irrespective of geographical location.

## Introduction

Infection with dengue virus (DENV) is an ever-increasing public health issue of global concern. DENV is endemic in more than 120 countries, with more than 40% of the world population at risk of DENV transmission (1, 2). Overall, it has been estimated that there are up to 100 million cases of dengue infection per year (3). Infections with any of the four different DENV serotypes can either be asymptomatic, as it is in more than 75% of the cases, or lead to clinical manifestations ranging from mild febrile illness (dengue fever, DF) to severe disease (dengue hemorrhagic fever, DHF) and shock syndrome with plasma leakage (dengue shock syndrome) (2, 4).

The role of T cells in severe dengue disease has been a topic of spirited debate and remains controversial. A role for T cells in the immunopathogenesis of the virus has been proposed based on the higher TNFα vs IFNγ production seen in DHF, and the suboptimal degranulation and cytokine production with heterologous peptides stimulation (5–7). Conversely, several studies have proposed a protective role for DENV-specific CD4^+^ and CD8^+^ T cells with increased frequency in subclinical infection relative to severe disease, effective antiviral activity of CD8^+^ T cells during secondary DENV infection and HLA-restricted protective responses associated with specific phenotypes and polyfunctionality (8–12). The general consensus is that in addition to antibodies, CD8^+^ and CD4^+^ T cell responses should be taken into consideration in accounting for a complete DENV-specific adaptive immunity.

Several issues need to be addressed to enable the global assessment of T cell responses. First, accurate assessment of responses requires the identification of a sufficient number of T cell epitopes. We and others (12–16) have studied responses observed in the general population in endemic areas to define the epitope repertoire associated with at least partial natural immunity. Second, geographic variations in HLA frequencies underscore the necessity of defining sets of epitopes that can elicit responses restricted by the most representative and common HLA alleles worldwide. Thus, it is important that epitope identification studies are conducted in different geographical locations, to enable identification of epitopes restricted by a diverse set of HLA molecules.

We previously determined the repertoire of DENV-specific CD8^+^ T cell responses in the endemic region of Colombo (Sri Lanka) and reported that different HLA alleles are associated with differential magnitudes of response (13). When the repertoire of CD8^+^ T cell responses was assessed in a different cohort of donors derived from the endemic region of Managua, Nicaragua, we observed a very similar HLA-associated hierarchy of responses (11).

Similarly, when the repertoire of DENV-specific CD4^+^ T cell responses in the Colombo region of Sri Lanka was determined, different HLA-DRB1 alleles were associated with different magnitudes of responses (12). In this study, we examined the DENV-specific CD4^+^ T cell responses in the Managua region and asked whether HLA-specific CD4^+^ T cell responses in Nicaragua and Sri Lanka were also similarly correlated.

In addition to a repertoire of epitopes spanning the most common HLA types, it is important to be able to assess responses directly *ex vivo*, so that the phenotypes are not altered. It is also critical to evaluate T cell responses in small sample volumes, as often only small volumes are available in large scale vaccine trials or study of severe DENV disease, especially in pediatric patients. To meet these challenges, we developed the megapool (MP) approach, which is based on a large numbers of peptides pooled and formulated *via* sequential lyophilization (17). DENV MPs for both CD8^+^ and CD4^+^ T cell epitopes have been previously reported (12, 14). The study of T cell responses in different settings and geographical locations is important in the definition of MPs that are truly global. Accordingly, we leveraged the combined information derived from our previous studies and this investigation of the CD4^+^ T cell repertoire in the Managua region to specifically design a comprehensive and widely applicable DENV CD4 MP that can detect T cell immune responses *ex vivo* independently of the HLA class II haplotypes represented in a given population.

## Materials and Methods

### Human Blood Samples

Blood bank samples from endemic areas were obtained from healthy adult blood donors from the Nicaraguan National Blood Center, Managua and from National Blood Center, Ministry of Health, Colombo, Sri Lanka, in an anonymous fashion, as previously described (11–14). We also studied a cohort of volunteers from Burlington, VT, Baltimore, MD, and Washington, DC, who were flavivirus naïve at the time of vaccination with a live-attenuated tetravalent DENV vaccine formulation (DLAV, TV005), as previously described (18, 19). Negative controls were healthy volunteers from San Diego, CA, who were tested and found to be seronegative for all DENV serotypes and yellow fever virus, and therefore defined in this study as “flavivirus naïve.” To verify the reactivity of the DENV epitopes, additional independent cohorts of DENV cases were selected in several endemic regions from Brazil, Singapore, and India. Peripheral blood mononuclear cells (PBMCs) were isolated by Ficoll density gradient centrifugation and frozen in fetal bovine serum supplemented with 10% DMSO (13, 14). Seropositivity to DENV was determined by testing for total anti-DENV antibodies by inhibition ELISA or antigen capture ELISA and modified microFRNT assay for neutralization titer (conducted at the National Virology Laboratory of the Nicaraguan Ministry of Health and the University of North Carolina, Chapel Hill, respectively), as previously described (20–22). All samples were collected and used following guidelines from the Institutional Review Boards (IRBs) of the La Jolla Institute for Allergy and Immunology (LIAI), the University of California, Berkeley, the Nicaraguan Ministry of Health, and the Medical Faculty, University of Colombo (serving as the National Institutes of Health-approved IRB for Genetech Research Institute) and clinical trials registered at ClinicalTrials.gov under registration numbers NCT01506570 and NCT01436422.

#### Brazil

Longitudinal samples were collected during the 2014 and 2015 outbreaks at the Hospital das Clinicas Universidade de São Paulo with acute and convalescent dengue specimens stored in the sample repository. The infecting DENV serotype was determined in all patients by RT-PCR. Samples obtained at least 1 month after the onset of acute dengue symptoms were made available for this study. All participants signed the IRB-approved informed consent document (IRB document 0652/09, CAPPesq, Hospital das Clínicas, Universidade de São Paulo).

#### Singapore

A cohort of DF cases was established at Tan Tock Seng Hospital enrolling patients with suspected DENV. DENV diagnosis was confirmed by the detection of DENV RNA by reverse transcription polymerase chain reaction, or of NS1 antigen by enzyme-linked immunosorbent assay (Bio-Rad), as previously described (9). The 16 samples used in this study were collected in the late convalescent phase 60–160 days after fever onset and all participants gave informed consent. Collection of PBMCs was approved by the Singapore National Healthcare Group ethical review board (DSRB 2008/00293).

#### India

Healthy adults were recruited into the study after advertisement by the Indian Institute of Science (IISc) and the National Institute of Mental Health and Neurosciences (NIMHANS), both in Bengaluru, Karnataka State, India, as part of a study on Japanese encephalitis (JE) and JE vaccine. The study was conducted according to the principles of the Declaration of Helsinki. All participants gave written informed consent and the protocol was approved by the IISc Institutional Human Ethics Committee (ref 5/2011). Previous exposure to DENV was determined by neutralization titers against all four serotypes, as previously described (23).

### HLA Typing and Phenotype Frequency Calculations

Donors were HLA typed by an ASHI-accredited laboratory at Murdoch University (Western Australia). HLA typing was performed for Class I (HLA A; B; C) and Class II (DQA1; DQB1, DRB1 3, 4, 5; DPB1), as previously described (12, 19, 24). Average phenotype frequencies for individual DRB1 alleles in the worldwide population are based on data available at DbMHC [NCBI (25)] and allelefrequencies.net (26), and were calculated as previously described (27–29). Briefly, for each HLA allele, an average gene frequency (gf) across Europe, North Africa, North-East Asia, South Pacific (Australia and Oceania), Hispanic North and South America, American Indian, South-East Asia, South-West Asia, and Sub-Saharan Africa populations was calculated, and an average phenotypic frequency (pf) was then derived utilizing the binomial distribution formula: pf = 1 − (1 − Σgf)^2^.

### MHC Class II-Binding Predictions and Peptide Selection

A set of DENV peptides predicted to bind various HLA-DRB1 allelic variants (DRB1*01:01, 01:02, 03:01, 04:03, 04:07, 07:01, 08:02, 09:01, 11:01, 11:04, 13:01, 14:02, 14:06, 15:01) was chosen according to the criteria described in the Section “Results” to assess DENV-specific HLA-restricted CD4^+^ T cell responses. Fifteen-mer peptides from all four serotypes were selected based on their predicted binding affinity to the selected MHC class II molecules (12, 30, 31). This resulted in the synthesis of more than 1,900 predicted HLA-DRB1-binding peptides (Mimotopes, VIC, Australia). Of a total of 1,931 peptides tested, 398 (21%) were predicted to bind two or more DRB1 alleles, and only 138 (7%) were predicted to bind to three or more. For screening studies, the class II peptides were combined into pools of approximately 20 individual peptides, according to their predicted HLA restriction, resulting in approximately 7 pools per HLA allele. Table S1 in Supplementary Material lists the number of peptides synthetized for each allele and serotype. For MP generation, each individual peptide was resuspended in 40 mg/ml DMSO. All resuspended peptides were then pooled and re-lyophilized. The resulting lyocake was resuspended in DMSO corresponding to a concentration of 1 mg/ml of each individual peptide and then diluted 1:4 in distilled water. Similarly, a set of 122 previously defined CMV- and EBV-specific peptides was resuspended as a MP (1 mg/ml) and used as a control pool in the intracellular cytokine staining (ICS) experiments (17).

### *In Vitro* Expansion of DENV-Specific T Cells

CD4^+^ T cells isolated from thawed PBMCs by magnetic bead negative selection and autologous antigen-presenting cells (APCs) were cultured in RPMI 1640 (Omega Scientific) supplemented with 5% human serum (Cellgro) at a 2:1 ratio and a density of 2 × 10^6^ and 1 × 10^6^ cells/ml, respectively, in 24-well plates (BD Biosciences). Cells were stimulated with DENV-specific peptide pools (averaging 20 peptides per pool) and incubated at 37°C in 5% CO_2_. IL-2 (10 U/ml; eBioscience) was added 4, 7, and 11 days after initial antigenic stimulation. Cells were harvested and screened for individual DENV-specific peptide reactivity on day 14.

### IFNγ ELISPOT Assay

Peripheral blood mononuclear cells (5 × 10^4^) were incubated in triplicates with 0.1 ml complete RPMI 1640 in the presence of HLA-matched peptide pools and individual peptides (2 µg/ml) after 14 days of *in vitro* expansion. After 20 h of incubation at 37°C, cells were incubated with biotinylated IFNγ mAb (mAb 7-B6-1 Mabtech, Stockholm, Sweden) for 2 h and developed as previously described (10, 13).

### Flow Cytometry

For ICS, PBMCs were cultured in the presence DENV CD4 MPs and CMV/EBV MP (1–2 µg/ml) for 2 h prior to the addition of GolgiPlug containing brefeldin A (BD Biosciences, San Diego, CA, USA) and further incubation for 4 h. At the end of the stimulation, cells were stained for surface markers, then permeabilized, and finally stained for intracellular markers, and then analyzed as previously described (13). Gating strategy is summarized in Figure S1 in Supplementary Material.

## Results

### Analysis of HLA Frequencies in Nicaragua and Selection of Target Alleles

Previous studies in the DENV endemic region of Colombo, Sri Lanka, analyzed a total of 16 HLA-DRB1 alleles present at frequencies of ≥5% in that population (12). Here, we expanded our analysis of HLA-DRB1-restricted responses to the Managua region in Nicaragua, which is also endemic for DENV infections. As a first step, we performed HLA typing on PBMCs from 334 healthy blood bank donors. The frequency of different HLA-DRB1 alleles was analyzed to identify alleles frequent in Nicaragua and not covered by the previous Sri Lanka study.

**Figure 1:**
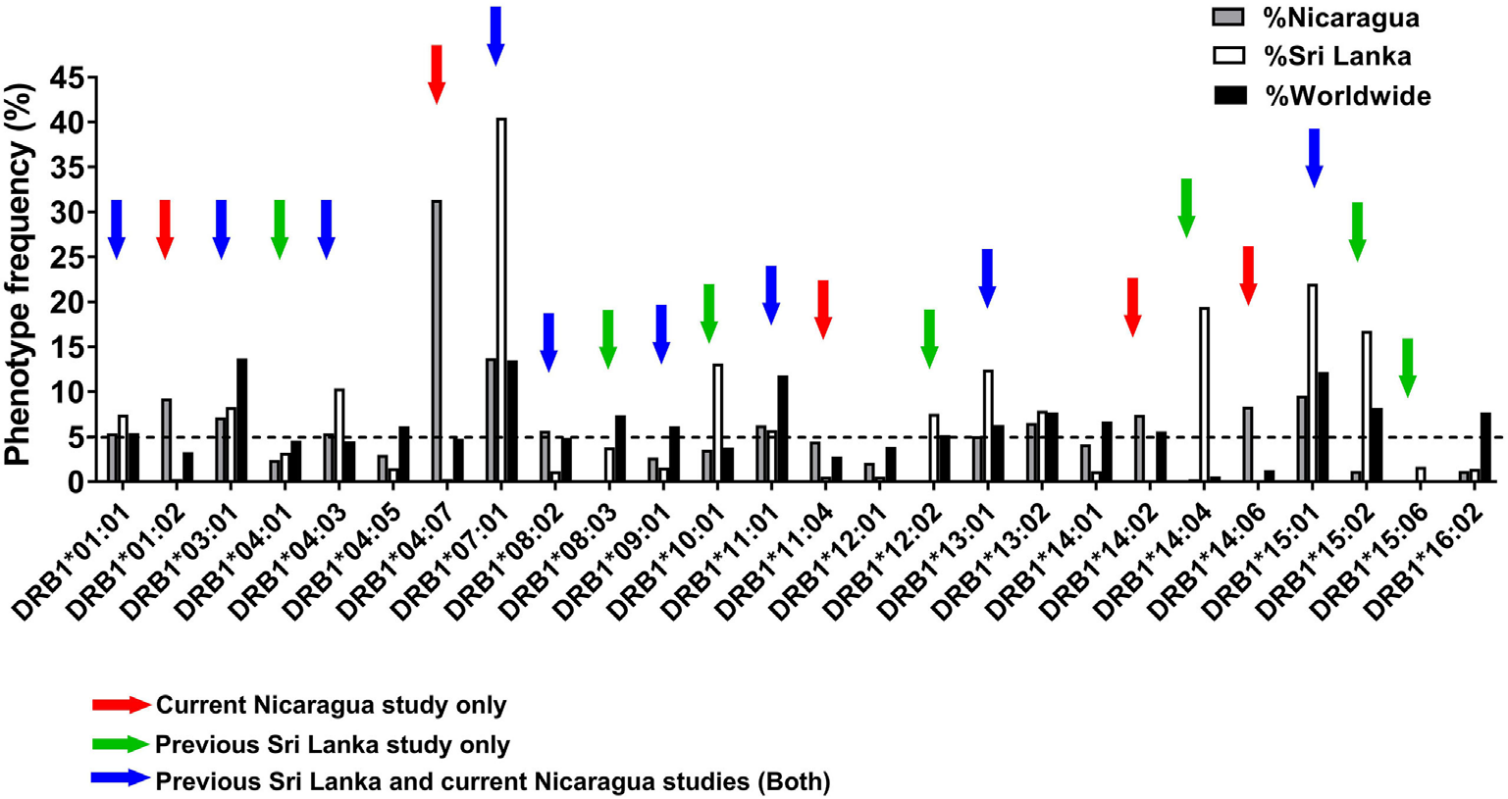
Phenotypic frequency of different HLA-DRB1 allelic variants in Nicaragua, Sri Lanka, and worldwide populations. The HLA-DRB1 phenotypic frequency from 334 Nicaraguan donors (gray bars) were compared to frequencies in Sri Lanka donors [(12); white bars] and worldwide (calculated as described in Section “Materials and Methods”; black bars). Red arrows indicate DRB1 alleles studied for epitope identification in this study only (not previously studied in Sri Lanka). Green arrows indicate DRB1 alleles not considered for epitope identification in this study, but previously addressed by the Sri Lankan studies. Blue arrows indicate DRB1 alleles studied for epitope identification in this study, and also previously addressed in the Sri Lankan studies.

Figure 1 shows the phenotypic frequency of alleles found at a frequency of 5% or greater in the Nicaraguan cohort (Figure 1, gray bars) and in the previously assessed Sri Lanka cohort (Figure 1, white bars) (12). The average allele frequency in the global worldwide population, calculated as described in the Section “Materials and Methods,” is also shown for comparison (Figure 1, black bars). To expand coverage of the Nicaraguan cohort, five HLA-DRB1 alleles that were not previously studied, but present at frequency ≥5% in Nicaragua (Figure 1, red arrows), were targeted for the epitope identification studies described below.

To examine potential differences or similarities in HLA-DRB1-associated responses in Nicaragua and Sri Lanka, we selected nine HLA-DRB1 alleles previously studied in Sri Lanka which were also present at a frequency ≥5% in Nicaragua (blue arrows in Figure 1). An additional seven HLA-DRB1 alleles for which the epitope repertoire was previously studied in Sri Lanka (green arrows in Figure 1) were not further analyzed in this study. Overall, the 21 HLA-DRB1 alleles targeted for epitope identification studies by this and previous Sri Lankan study (marked by the colored arrows in Figure 1) cover 81.7% of alleles with phenotype frequency ≥5% worldwide. Combined, these alleles afford for 92% phenotypic coverage worldwide (i.e., 92% of individuals express at least one of these alleles).

### Overall CD4 Reactivity in the Nicaragua Cohort for the HLA-DRB1 Alleles Studied

Previous experiments analyzed CD4^+^ T cell responses restricted by various HLA-DRB1 alleles (12) in a cohort of Sri Lankan donors. Potential epitopes from each of the four DENV serotypes predicted to bind at least 1 of 15 HLA-DRB1 alleles were selected, as previously described (12). An overall summary of the number of predicted binders for each DENV serotype and HLA allele is presented in Table S1 in Supplementary Material. For each HLA-DRB1 allelic variant considered, approximately 130 predicted peptides were tested in HLA-DRB1 matched donors, also as previously described (10).

CD4^+^ T cells purified through negative selection using magnetic beads were cultured with autologous APCs and stimulated with HLA-matched DENV-specific pools. On day 14, cells were harvested and screened for reactivity against individual DENV-specific peptides in an IFNγ ELISPOT assay. A total of 4–10 donors for each of the HLA-DRB1 allelic variants were tested. A complete listing of all peptides tested, including response frequency and HLA restriction has been submitted to the Immune Epitope Database (IEDB)^1^^,^2^^ and the Immport database^3^ (Submission ID: SDY1109). The overall reactivity as a function of the different HLA alleles and different proteins is shown in Table 1. The average magnitude of responses for each allele (total magnitude of responses detected for that allele corrected for the number of donors tested) was 13,764 ± 6,451 (IFNγ SFC/10^6^ PBMC) and the average number of epitopes/donor recognized in the context of each allele was 11 ± 4 epitopes/donor.

**Table 1:** CD4^+^ T cell dengue virus (DENV) immunoreactivity in peripheral blood mononuclear cells from the Nicaraguan cohort studied.

HLA DRB1* allele	Total response magnitude/donor	Average # of epitopes/donor	DENV proteins									
			C	prM	E	NS1	NS2A	NS2B	NS3	NS4A	NS4B	NS5
0101	11,300	6.6	7,191	172	0	292	324	556	2,343	0	422	0
0102	10,998	9.9	132	0	455	0	2,332	167	5,954	417	1,045	495
0301	8,557	9.5	0	0	162	3,054	495	751	1,500	0	1,941	654
0403	8,575	7.8	1,505	0	3,176	68	462	0	1,466	157	0	1,741
0407	15,958	13.5	6,441	0	2,033	636	514	0	5,856	0	64	414
0701	11,308	11.5	1,897	666	1,211	742	1,854	224	2,043	0	1,406	1,266
0802	5,758	9.8	1,124	126	275	0	0	619	1,020	62	0	2,532
0901	9,964	7.2	2,663	264	93	676	321	0	997	0	338	4,612
1101	25,412	18.7	9,658	1,236	1,774	0	2,836	90	3,747	915	251	4,905
1104	5,708	5.4	0	0	597	749	1,718	0	505	100	0	2,039
1301	17,888	13.7	3,565	0	123	0	3,649	0	6,380	560	916	2,694
1402	20,681	17.1	12,914	0	3,422	0	216	0	2,648	0	137	1,345
1406	24,365	19.7	8,162	0	2,658	936	207	0	8,159	0	288	3,955
1501	16,227	11.8	1,755	951	3,165	0	439	0	5,755	2,335	1,107	719
Average	13,764	11.6	60,178	3,805	19,900	7,439	15,367	2,407	48,374	4,546	8,485	29,385
SD	6,451	4.5	30%	2%	10%	4%	8%	1%	24%	2%	4%	15%

### Similar Patterns of Protein Immunodominance and Higher Magnitude of DENV-Specific Responses in the Nicaraguan Cohort

**Figure 2:**
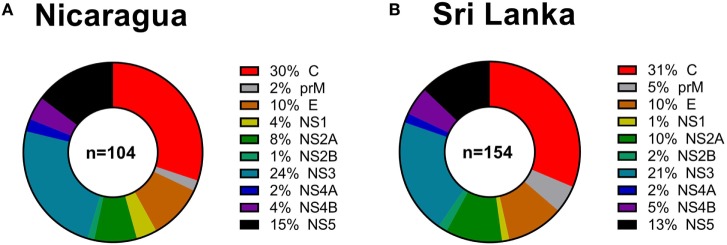
Patterns of protein immunodominance of CD4^+^ T cells responses in Nicaragua and Sri Lanka. Pie charts indicate the percentage of total magnitude of responses per protein observed in 104 Nicaragua **(A)** and 154 Sri Lanka **(B)** donors tested using identical methodology. Data from the Sri Lanka donors were published previously (12) and are shown here for reference purposes only.

Next, we considered the pattern of epitope immunoreactivity in the Nicaraguan cohort as a function of location within the DENV genome, and in comparison to the pattern previously observed in the Sri Lankan cohort (12). Pie charts in Figure 2 show data from the Nicaragua (Figure 2A) and Sri Lankan (Figure 2B) cohorts. The pattern of immunodominance in the two cohorts is remarkably similar, with the C, NS5, and NS3 antigens being most dominant in both, and accounting for 30 vs 31%, 24 vs 21%, and 15 vs 13% of the total response in the Nicaraguan and Sri Lankan cohorts, respectively.

We further compared the magnitude of CD4^+^ T cell responses in the Nicaraguan cohort to that previously observed in Sri Lankan donors (Table 2). When the 9 alleles tested in both cohorts are considered, the average magnitude of Nicaraguan responses was 12,776 ± 6,069 (IFNγ SFC/10^6^ PBMC), while in the Sri Lankan study the average magnitude was 6,800 ± 4,288 (IFNγ SFC/10^6^ PBMC) (Table 2). The response observed in the Nicaraguan cohort was almost twofold higher than in the Sri Lankan cohort (*p* = 0.0315 in a two-tailed Mann–Whitney test). A similar trend was observed when comparing the number of epitopes recognized for each allele in the two cohorts (10 ± 4 vs 6 ± 3; *p* = ns).

**Table 2:** Comparison of CD4^+^ T cells dengue virus immunoreactivity between Nicaragua and Sri Lanka.

	Nicaragua		Sri Lanka	
	Total magnitude	Average epitopes per donor	Total magnitude	Average epitopes per donor
DRB1*0101	11,300	6.6	5,447	6.80
DRB1*0301	8,557	9.5	2,064	3.1
DRB1*0403	8,575	7.8	3,668	4.6
DRB1*0701	11,308	11.5	11,350	9.7
DRB1*0802	5,758	9.8	3,761	2.9
DRB1*0901	9,964	7.2	10,039	10.6
DRB1*1101	25,412	18.7	1,659	2.2
DRB1*1301	17,888	13.7	11,732	10.4
DRB1*1501	16,227	11.8	11,476	7.8

Average	12,776	10.7	6,800	6.5
SD	6,069	3.8	4,288	3.4

*Data from the Sri Lanka donors were published previously (12) and are shown here for reference purpose only*.

### Differences in Patterns of HLA-Linked Immunodominance of CD4 Reactivity in Sri Lanka and Nicaragua Cohorts

**Figure 3:**
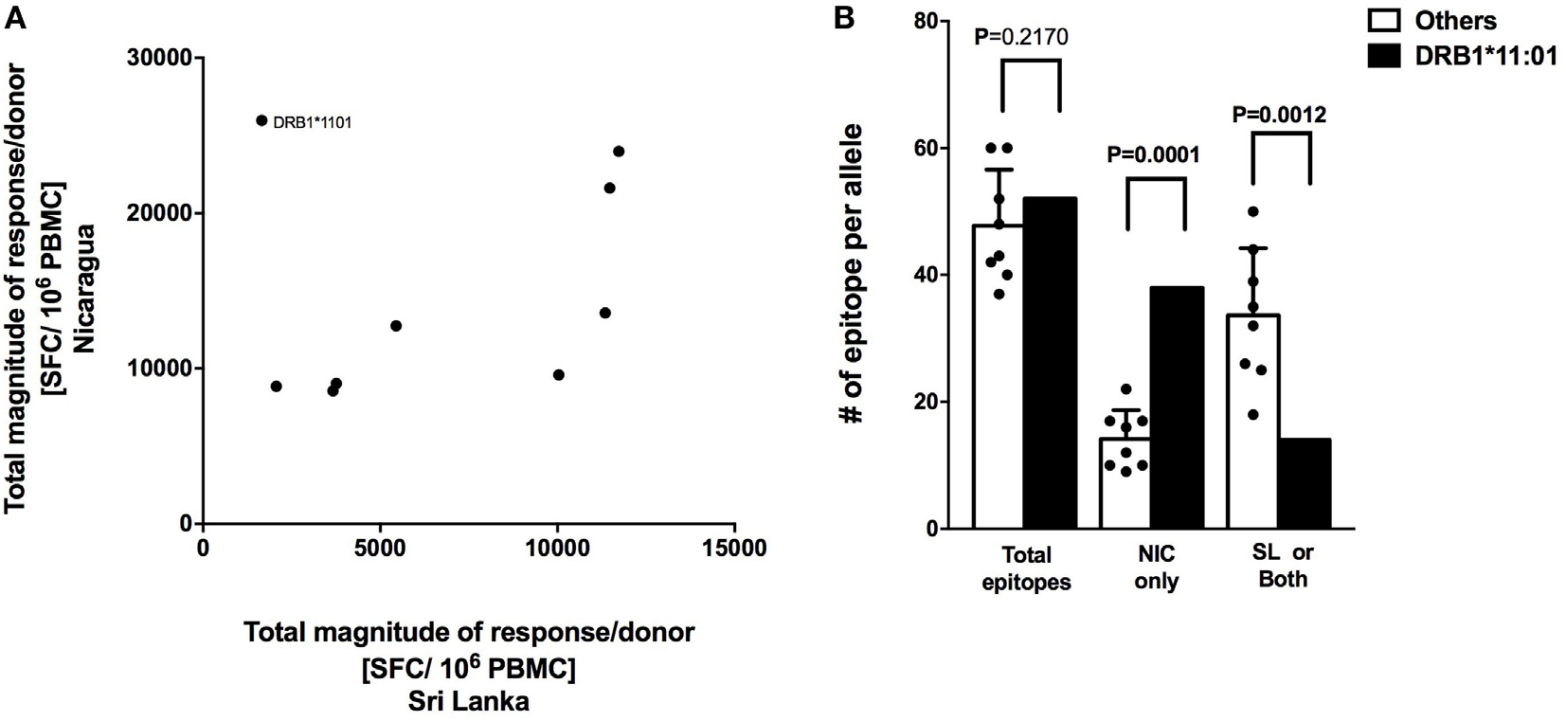
Correlation of HLA-restricted CD4^+^ T cells responses in Nicaragua and Sri Lanka cohorts. **(A)** Correlation of total responses magnitude per donor as a function of restricting HLA molecule, between Nicaraguan and Sri Lankan cohorts (*R*^2^ = 0.07291, *p* = 0.4823; if HLA-DRB1*11.01 is removed, *R*^2^ = 0.5537, *p* = 0.0343). **(B)** Comparison of the number of epitopes detected in the case of the HLA-DRB1*11:01 allele and in other eight HLA-DRB1 alleles (*01:01, *03:01, *04:03, *07:01, *08:02, *09:01, *13:01, and *15:01) considered in the study using one-sample *t* test. Bars refer to total epitopes, epitopes detected only in Nicaragua (NIC), and epitopes detected in Sri Lanka (SL) only or both cohorts.

CD8^+^ T cell responses measured in Sri Lankan and Nicaraguan healthy blood donor cohorts are associated with a very similar HLA-associated hierarchy of responses (11). The total response magnitude per donor for each HLA-DRB1 alleles analyzed in this study was then compared to the total magnitude of the same HLA-DRB1 previously tested in a Sri Lankan cohort using the (12). No correlation of HLA-DRB1 restriction and magnitude of responses was apparent (Figure 3A; *R*^2^ = 0.07291, *p* = 0.4823). Removing the “outlier” HLA-DRB1*11:01 allele with higher total responses magnitude per donor in the Nicaraguan cohort compared to the Sri Lankan cohort led to a significant, albeit weak correlation (Figure 3A; *R*^2^ = 0.5537, *p* = 0.0343). Even in this case, the correlation is much less than that previously observed in CD8^+^ T cell responses (*R*^2^ = 0.98) (11). The mechanism for this poor (or lack of) correlation was further investigated in the case of the HLA-DRB1*11:01 “outlier.”

We specifically tested whether the higher HLA-DRB1*11:01 CD4^+^ T cell responses in the Nicaraguan cohort were due to recognition of the same epitopes with higher magnitude of responses or, alternatively, whether the greater response to HLA-DRB1*11:01 was due to recognition of a new and/or broader set of epitopes. When looking at the total number of epitopes identified in the 2 cohorts, 52 total HLA-DRB1*11:01 epitopes and on average 48 (range 39–60) total epitopes restricted to the other 8 HLA-DRB1 alleles were identified. Therefore, in terms of total number of epitopes, DRB1*11:01 does not seem to recognize more epitopes respect to the other alleles in this study. We then looked and the number of epitopes recognized only in Nicaragua, or only in Sri Lanka or both populations. Out of 52 total HLA-DRB1*11:01 epitopes, 38 (73.1%) were recognized uniquely by CD4^+^ T cells from Nicaragua subjects, and only 14 (27.9%) were recognized in both populations or only the Sri Lankan cohort (Figure 3B).

In conclusion, when including all alleles studied, we did not observe a clear HLA-DRB1 response hierarchy of CD4^+^ T cell responses, which is in contrast to what we have previously detected at the level of CD8^+^ T cell responses (11). Our results also suggest that the HLA-DRB1*11:01 allele was able to recognize a broader array of epitopes presented only in the Nicaraguan cohort, in contrast to what was observed for the other alleles, where epitope-specific responses were detected in both cohorts (11).

### Epitope Selection for a Global DENV CD4 MP

Pools of large numbers of epitopes, generated by sequential lyophilization (17), have been utilized as a tool to globally characterize both CD4^+^ and CD8^+^ T cell responses. These MPs have been validated in a number of contexts, including allergies (32), latent tuberculosis infection (33), tetanus/pertussis vaccination (34, 35), and DENV immunity (12, 14). We previously constructed CD4 DENV MP based on epitopes recognized by CD4^+^ T cells from Sri Lankan subjects (12). Here, we reevaluated the DENV CD4 MP by studying a broader set of epitopes than previously examined, including epitopes restricted by additional HLA alleles deriving from Nicaraguan subjects.

**Figure 4:**
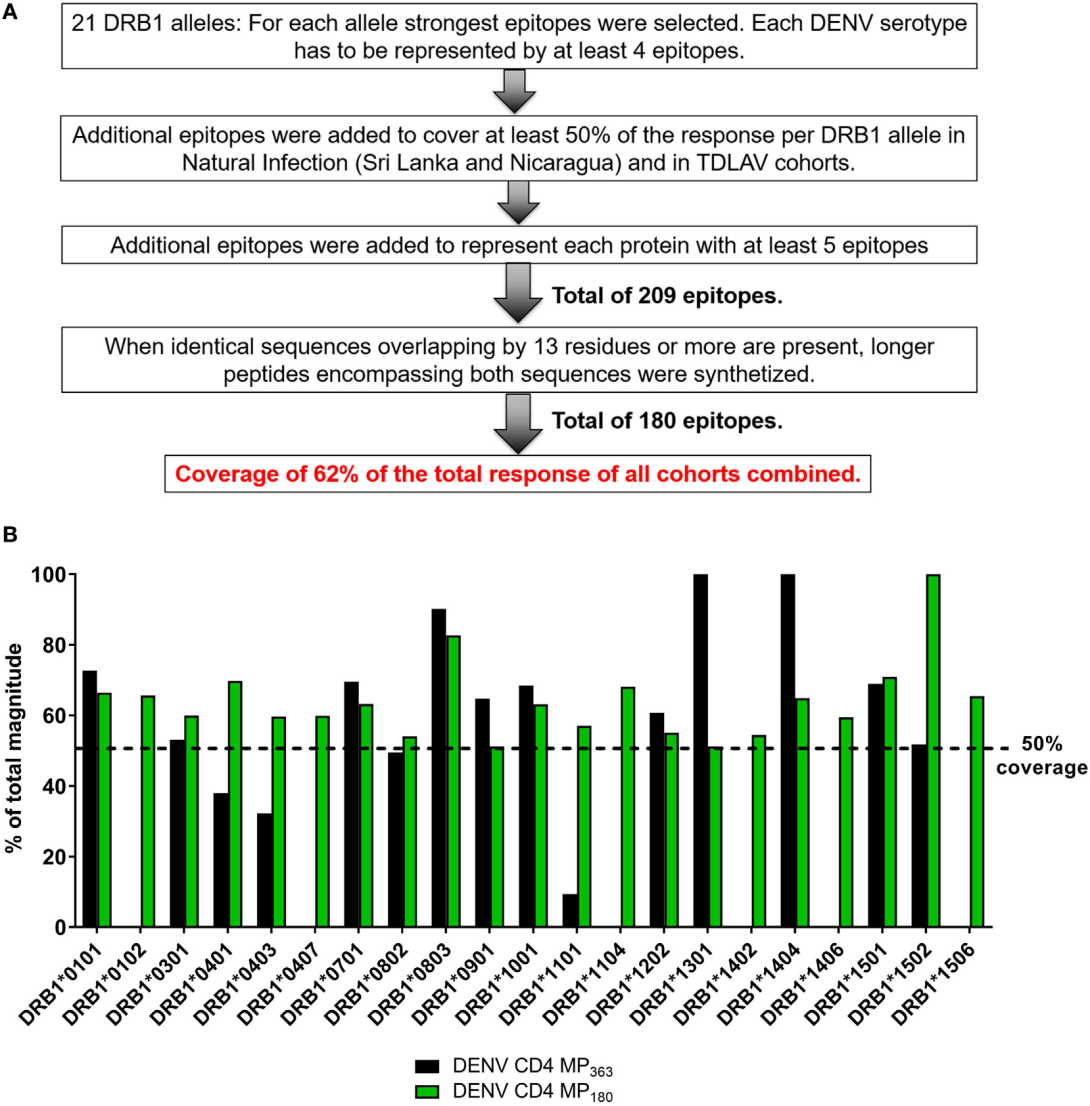
Characteristics of dengue virus (DENV) CD4 MP_180_. **(A)** Strategy used to select the epitopes composing the DENV CD4 MP_180_. **(B)** Comparison of HLA coverage in terms of magnitude of responses for epitopes belonging to the two DENV CD4 MPs.

The previously published MP (DENV CD4 MP_363_) was composed of 363 epitopes defined in the Sri Lankan cohort (12). Here, we took a more structured approach to derive a new DENV CD4 MP (Figure 4A). First, we selected the highest response magnitude epitopes, considering responses in both the Sri Lankan and Nicaraguan cohorts, but also a previously described cohort of individuals immunized with a Tetravalent Dengue Live-Attenuated Vaccine (TV005) (19), until coverage of at least 50% of the total response was reached for each of the 21 HLA-DRB1 alleles identified above. If needed, additional epitopes were selected to ensure that each DENV serotype was represented by at least four epitopes for each HLA-DRB1 allele (some peptides are conserved in multiple serotypes, and thus fewer epitopes were required to meet this criteria). Additional epitopes were also selected to ensure that each DENV protein was represented by at least five epitopes. This process resulted in selection of a total of 209 epitopes. In instances where sequences overlapped by at least 13 residues, longer peptides encompassing both sequences were synthetized. This resulted in a final selection of 180 epitopes, which allowed coverage of 62% of the total responses of all cohorts combined.

The HLA coverage and magnitude of responses of the new optimized DENV CD4 MP_180_ was compared to the previous DENV CD4 MP_363_ (Figure 4B). Across the different HLA alleles, the DENV CD4 MP_180_ provided an average coverage of 62.4% (±13.6) of the total response, which is higher and more consistent than the previous DENV CD4 MP_363_ (42.0 ± 35.7%). DENV serotype coverage and protein representation had very similar patterns of coverage, with both DENV CD4 MPs providing fairly balanced coverage across the four serotypes (Figure S2A in Supplementary Material) and very similar patterns of protein representation (Figure S2B in Supplementary Material).

### The DENV CD4 MP_180_ Affords Higher and More Consistent Coverage in Different Donor Cohorts

**Figure 5:**
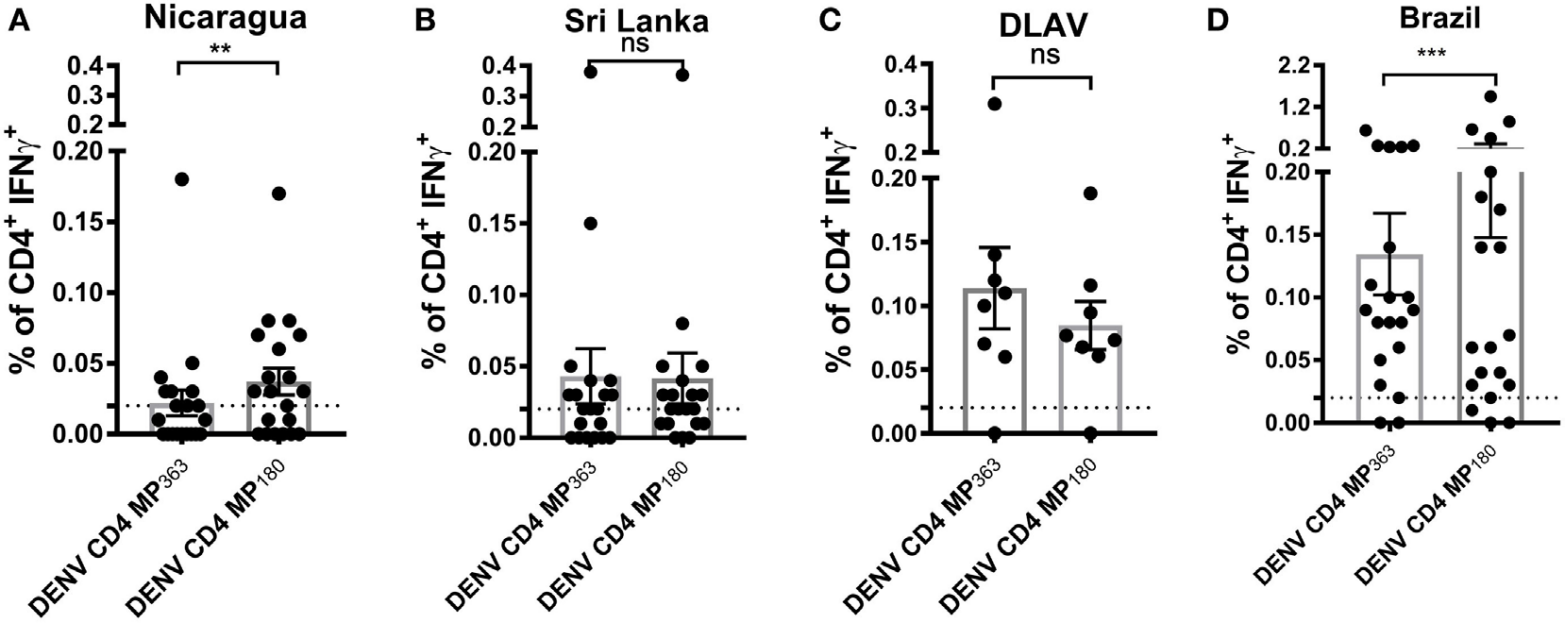
Comparison of CD4^+^ T cell reactivity to dengue virus (DENV) CD4 MP_363_ and DENV CD4 MP_180_. Percentage of CD4^+^ T cells producing IFNγ upon stimulation with the two DENV CD4 MPs in Nicaraguan (*n* = 20) **(A)**, Sri Lankan (*n* = 20) **(B)** DLAV (*n* = 8) **(C)**, and Brazilian (*n* = 20) **(D)** donor cohorts. Data are expressed as mean ± SEM. Statistical analyses were performed using two-tailed Wilcoxon test. *p < 0.05; **p < 0.01; ns, not significant. The dashed lines at 0.02% represent cutoff of positivity.

To assess the capacity of the DENV CD4 MP_180_ to detect T cell responses *ex vivo* from DENV exposed individuals, the DENV CD4 MPs were next tested in ICS assays utilizing PBMCs from Nicaraguan and Sri Lankan donor cohorts, as well as a cohort of donors vaccinated with TV005 (19) (Figure 5). As expected, the DENV CD4 MP_180_ performed better in the Nicaraguan cohort. Specifically, significant reactivity was detected in 6 out of 20 donors for the DENV CD4 MP_363_ and 11 out of 20 donors for the DENV CD4 MP_180_. The overall magnitude of response between the two MPs is significant by a two-tailed Wilcoxon test (Figure 5A). We did not expect to see a significant difference in reactivity in the Sri Lankan cohort, as Sri Lankan epitopes were contained in both DENV CD4 MPs. Indeed, in the Sri Lankan cohort, significant reactivity was detected in 8 out of 20 donors for both DENV CD4 MPs, with no significant difference in the magnitude of responses (Figure 5B). Similarly, TV005-induced responses were detected in seven out of eight donors for both DENV CD4 MPs, with no significant difference in response magnitude (Figure 5C).

Next, we were interested to compare the performance of both DENV CD4 MP’s in a cohort that was not originally used to perform epitope identification. Accordingly, both pools were tested in a Brazilian cohort. Interestingly, also in a cohort that was not used to define the epitopes the MP180 pool works significantly better when compared to the previously defined CD4 MP, confirming the better performance of DENV CD4 MP180 when compared to DENV CD4 MP363 (Figure 5D).

**Figure 6:**
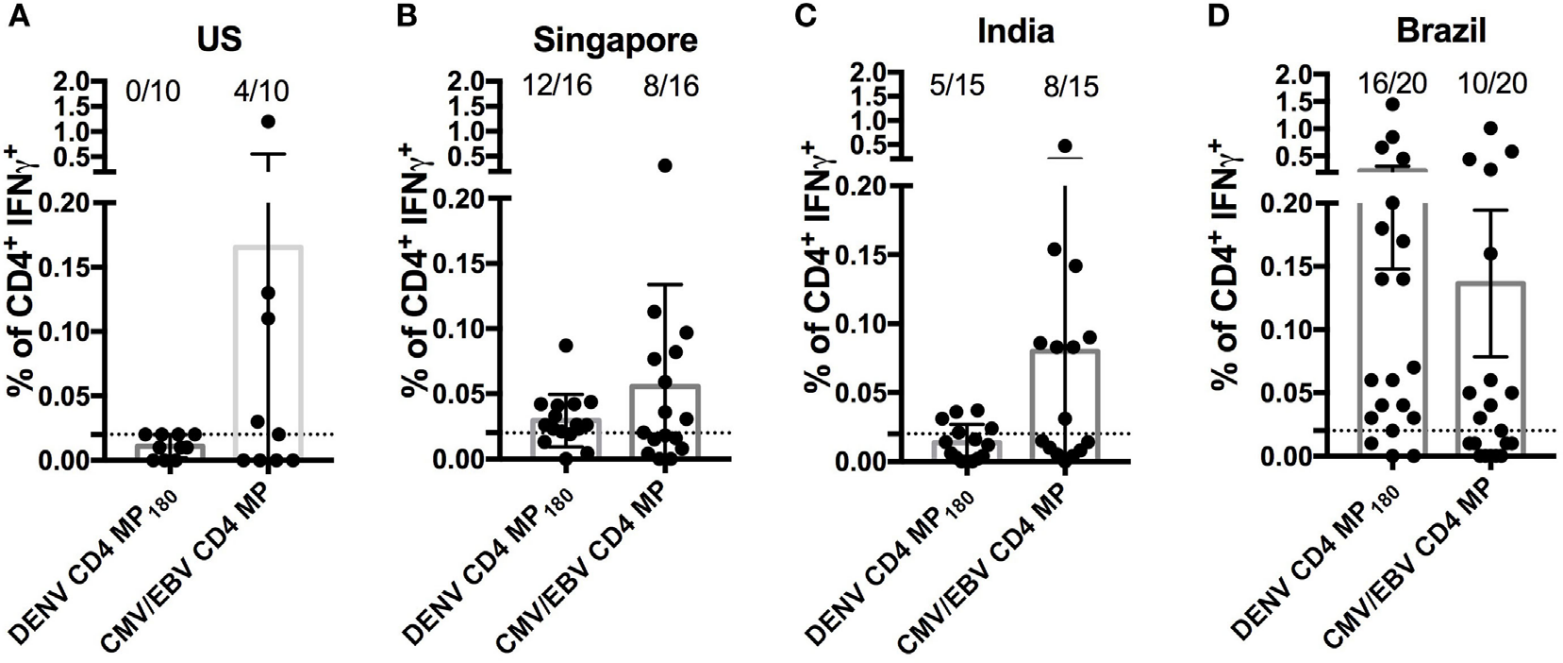
Assessment of dengue virus (DENV) CD4 MP_180_ reactivity in diverse geographical locations. Percentage of CD4^+^ T cells producing IFNγ upon stimulation with DENV CD4 MP_180_ in flavivirus naïve (*n* = 10) **(A)** and DENV exposed Singaporean (*n* = 16) **(B)**, Indian (*n* = 9) **(C)**, and Brazilian (*n* = 20) **(D)** donor cohorts. CMV/EBV CD4 MP stimulation was used as a control. To allow for comparison of the responses frequencies against the CMV/EBV and the dengue-specific response in all new cohorts the data showing response frequencies against the DENV CD4 MP_180_ in the Brazilian cohort from Figure 5D has been re-plotted in Figure 6D. Data are expressed as mean ± SEM, and the dashed lines at 0.02% represent the cutoff of positivity.

Finally, we asked whether the DENV CD4 MP_180_ was capable of globally capturing DENV reactivity when multiple cohorts from different geographical locations were considered. We specifically considered locations that are endemic for DENV infections and that had not provided samples for our epitope identification studies. Accordingly, we analyzed responses in PBMCs derived from Brazilian, Singaporean, and Indian cohorts. As a negative control, we assayed healthy volunteers from San Diego who had been tested and found to be seronegative for all DENV serotypes and yellow fever virus (“flavivirus naïve” controls). As a positive control, we included a previously described CMV/EBV MP (17) (Figure 6).

As expected, flavivirus naïve donors did not show reactivity to DENV CD4 MP_180_. Also, as expected, all the cohorts showed similar frequencies of reactivity, in the 45–55% range, against the CMV/EBV MP (Figure 6). DENV exposed cohorts were associated with a range of reactivity between 33 and 75% against DENV CD4 MP_180_. When frequencies and magnitude of response between cohorts were compared, magnitude but not frequency of response from Brazil was significantly higher compared to all other cohorts (Table S2 in Supplementary Material). Since the Brazilian cohort was not used to define the epitopes included in MP180, the most likely explanation is that these differences are due infection history and not MP composition. Nevertheless, the data should be interpreted with this caveat in mind.

## Discussion

In this study, we analyzed the epitopes recognized by DENV-specific CD4^+^ T cells in the context of different HLA alleles in the general population of Nicaragua, where DENV is highly endemic. Comparison of the patterns of responses to those previously defined using identical methodology in the Colombo endemic area of Sri Lanka revealed a striking similarity in the overall pattern of recognition at the antigenic level (12). Using the knowledge derived from the combined analysis in the two locations, we derived a MP of epitopes (DENV CD4 MP_180_) and showed that it can be applied to the study of DENV responses in PBMC samples derived from disparate geographical locations.

Several lines of evidence indicate that T cells play an important role in the control of DENV infection, disease pathogenesis, and responses to vaccination (36, 37). This suggests that, in parallel with antibody response, T cell responses should be measured and analyzed to achieve a complete assessment of DENV-specific adaptive immunity. In addition, peripheral CD4^+^ T cells probably contain a fraction of circulating memory follicular T helper cells that are relevant for B cell differentiation and the production of neutralizing antibodies (38, 39). Since DENV infection and disease is a worldwide and ever-increasing problem, a global approach to assess T cell responses in different human populations is required. While several studies have analyzed CD8^+^ T cell responses against DENV (11, 13, 15, 40, 41), less information is available on their CD4^+^ T cell counterparts (12, 42). In this context, investigating DENV-specific HLA-restricted CD4^+^ T cell epitopes recognized in different DENV endemic populations and restricted by a broad variety of HLAs is necessary to evaluate potential genetic and geographical factors affecting DENV reactivity.

Our strategy expands previous studies on HLA-DRB1 locus-specific responses in the DENV endemic region of Colombo, Sri Lanka (12), to increase the HLA-DRB1 coverage from 16 to 21 of the most common alleles. We estimate that together these alleles provide phenotypic coverage of 92% of individuals in the general worldwide population, thus accomplishing our goal of enabling global coverage of the HLA-DRB1 locus. This statement should be interpreted with two caveats in mind: first, certain alleles not particularly common on a worldwide basis might be common in particular populations. In these cases, additional epitope identification studies may be required to fill gaps in coverage for a specific population. Second, our strategy does not include the DRB3/4/5, DP, and DQ loci. We have previously shown that epitope predictions performed on the main DRB1 molecules should be able to cover approximately 50% of the total CD4^+^ T cell response (43). Despite this evidence, future studies are planned to expand the coverage of DENV CD4^+^ T cell epitopes by considering those loci. Third, we note that our assignment of these responses to the HLA DRB1 locus is based on binding predictions and correspondence with donor HLA type. However, it is possible, given the well-known crossreactivity of HLA class II, that some of the response is in fact due to coexpressed DP or DQ specificities. However, we believe that this collection of epitopes will provide wide coverage based on the fact that different HLA class II molecules exhibit significant inter- and intra-loci repertoire overlap (28, 29, 44, 45) and that our epitope sets were tested in various geographical locations. An issue of relevance for this study is the accuracy and reliability of epitope prediction software, the IEDB runs periodic benchmarking of its tools, the latest published benchmarking for HLA class II (31) found AUC values in the 0.622–0.818 range for the DRB1 alleles, the HLA class II molecules of interest for this study. However, it is certainly possible that differences in predictive capacity or performance may influence the results and should therefore be interpreted with this caveat in mind.

Nicaraguan DENV-specific CD4^+^ T cell responses preferentially targeted C, NS3, and NS5. This pattern is in agreement with what we previously observed in Sri Lanka (12), as well as what was also detected by Rivino et al. in DENV infected donors from Singapore (46), suggesting a similar protein source for immunodominant epitopes in different endemic areas. This finding is not only significant for the study of DENV CD4^+^ T cell immunity but also to the design and evaluation of vaccines. Indeed, this pattern of immunodominance is also observed in individuals vaccinated with an experimental tetravalent live-attenuated vaccine formulation (TV005) and the attenuated DENV2 virus (Tonga 74) used as a challenge strain in a DENV human challenge model (19, 24). Accordingly, we conclude that the patterns of immunodominance induced by vaccination with attenuated DENV vaccines should be able to mimic natural immunity regardless of geographical location.

The patterns of HLA-linked immunodominance previously observed at the level of CD8^+^ T cell responses (11) in the Sri Lankan and Nicaraguan cohorts were virtually indistinguishable. Here, at the level of HLA class II, we did not detect a significant correlation between response magnitudes between the two cohorts. A caveat to this is that our results were based on a limited number of HLA allelic variants. In pursuing this line of inquiry, we uncovered the unique case of the HLA-DRB1*11:01 allele, which exhibited much higher reactivity in the Nicaragua cohort as compared to the Sri Lanka cohort. Furthermore, most of the differences were related to recognition of a new set of epitopes recognized specifically in the Nicaragua cohort. The mechanism of this is as yet unclear. Strong T cell responses/donor relative to other alleles were observed in the case of HLA-DRB1*11:01, *14:02, and *14:06 alleles in the Nicaragua population. HLA-DRB1*11:01 recognized a new and broader set of epitopes that is unique to the cohort from Nicaragua when compared to previously identified epitope sets in Sri Lanka. In the case of HLA-DRB1*14:02+ and *14:06+ donors with similar strong responses, this comparison was not possible as those alleles are not present in the Sri Lanka populations (Figure 1).

Thus, it is possible that that the differences observed for HLA-DRB1*11:01 between the two populations could be observed for other alleles with similar magnitude of T cell response/donor. It is also possible that these differences may relate to the potential contribution of other loci in linkage disequilibrium with the HLA-DRB1 locus, and sharing some degree of repertoire overlap. The future studies of DENV CD4^+^ T cell epitopes restricted by HLA-DRB3/4/5, DP, and DQ loci will address this hypothesis.

Our original goal was to identify reagents and assay strategies to allow assessment of DENV CD4^+^ T cell responses regardless of geographical location in the specific context of DENV disease or vaccination. This first required the identification of a repertoire of epitopes spanning the most common HLA types. In addition, it was important to adopt methodologies amenable to direct *ex vivo* analysis to avoid altering T cell phenotypes and to sample-preserving approaches given that volume is often limiting in vaccine studies and pediatric cohorts. To meet these challenges, our group has developed the MP approach, based on large numbers of peptides pooled and formulated to take into account sequential lyophilization (17). In this study, we refined the previously described DENV CD4 MP_363_ by incorporating information from the Nicaraguan study and using a rational, optimized, epitope selection strategy targeting balanced HLA, DENV serotype, and protein composition coverage in the process of epitope selection. We validated this optimized DENV MP by showing that it can be used to assess DENV-specific CD4^+^ T cell responses across donors independently from HLA type, geographical region, or infecting serotype. It is apparent that different cohorts vary somewhat in reactivity to the MP180 pool. This differential reactivity might be due to insufficient coverage or due to the different history of DENV infection and burden of the disease. The DENV cohorts from Singapore and India (Figures 6B,C) are associated with relatively lower levels of secondary DENV infection (47, 48), as compared with the Brazilian cohort where secondary infections were more prevalent, reflecting a higher burden of infection (49, 50). We have previously shown that multiple exposures to DENV increase the magnitude of CD4^+^ T cell DENV-specific responses relative to primary infection suggesting that infection history can influence magnitude of DENV-specific T cell responses (10). Most importantly, we showed that the assessment can be performed directly *ex vivo* using ICS assays. This has multiple advantages compared to other assays such as ELISPOT. Gating strategies allow the exclusion of CD8^+^ T cell responses that might respond to nested MHC class I epitopes within the tested 15mer peptide. Furthermore, it will allow parallel phenotypic assessments across different studies and capture of antigen-specific T cells for transcriptomic analysis on a large scale. Future studies will address the secretion of multiple cytokines and marker expression revealing potential additional dengue-specific CD4^+^ T cell responses but also specific polarizations of T cell responses between the different cohorts.

## Ethics Statement

All samples were collected and used following guidelines from the Institutional Review Boards (IRBs) of the La Jolla Institute for Allergy and Immunology (LIAI), the University of California, Berkeley, the Nicaraguan Ministry of Health, and the Medical Faculty, University of Colombo (serving as the National Institutes of Health-approved IRB for Genetech Research Institute) and clinical trials registered at ClinicalTrials.gov under registration numbers NCT01506570 and NCT01436422. All Brazilian participants signed the IRB-approved informed consent document (IRB document 0652/09, CAPPesq, Hospital das Clínicas, Universidade de São Paulo). Collection of PBMCs from Singapore was approved by the Singapore National Healthcare Group ethical review board (DSRB 2008/00293). All Indian participants gave written, informed consent and the protocol was approved by the IISc Institutional Human Ethics Committee (ref 5/2011).

## Author Contributions

AG, MA, BL, PHoR, CC, CS, AM, PC, MC, QL, FT, AD, and LR performed experiments, reviewed data, and planned the experimental strategy. JS and BP performed bioinformatics analyses. EP and SM performed and coordinated HLA typing and related analysis. AB, APD, SD, ADdS, GN, CN, AMdS, PM, TS, VR, VS, JC, EK, LT, and EH collected samples and provided clinical information. AG, AS, and DW conceived and directed the study, and wrote the manuscript. All the authors have critically read and edited the manuscript.

## Conflict of Interest Statement

The authors declare that the research was conducted in the absence of any commercial or financial relationships that could be construed as a potential conflict of interest.

## Footnotes

^1^http://www.iedb.org.

^2^http://www.iedb.org/subID/1000719.

^3^http://www.immport.org.
