# Supplementary material for: Global Assessment of Dengue Virus-Specific CD4+ T Cell Responses in Dengue-Endemic Areas
Source: Front Immunol. 2017 Oct 13;8:1309. doi: 10.3389/fimmu.2017.01309 (PMC5646259; doi:10.3389/fimmu.2017.01309)
Supplement: Supplementary file 1 [file Data_Sheet_1.DOCX]

Supplementary Material

**Global assessment of dengue virus-specific CD4^+^ T cell responses in dengue-endemic areas**

**Alba Grifoni^1^, Michael A. Angelo^1^, Benjamin Lopez^1^, Patrick H. O’Rourke^1^, John Sidney^1^, Cristhiam Cerpas^2^, Angel Balmaseda^2^, Cassia G. T. Silveira^3^, Alvino Maestri ^3^, Priscilla R. Costa^3^, Anna Durbin^4^, Sean A. Diehl^5^, Elizabeth Phillips^6^, Simon Mallal^6^, Aruna D. De Silva^1,7^, Godwin Nchinda^8^, Celine Nkenfou^8^, Mathew H. Collins^9^, Aravinda M. de Silva^9^, Lim Mei Qiu^10^**, **Paul A. Macary^11^, Filippo Tatullo^12^, Tom Solomon^12,13^, Vijaya Satchidanandam^14^, Anita Desai^15^, Vasanthapram Ravi^15^,** **Josefina Coloma^16^, Lance Turtle^12,13^, Laura Rivino^10^, Esper G. Kallas^3^, Bjoern Peters^1^, Eva Harris^16^, Alessandro Sette^1^ and Daniela Weiskopf ^1*^**

*** Correspondence:**Dr. Daniela Weiskopf
[daniela@lji.org](mailto:daniela@lji.org)

**Supplemental table 1. List of all the peptides analyzed in this study based on HLA-DRB1 restriction and DENV serotype.**

|  | DENV1 | DENV2 | DENV3 | DENV4 | conserved (80% homology) | **Total peptides per HLA** | |
| --- | --- | --- | --- | --- | --- | --- | --- |
| DRB1*0101 | 26 | 17 | 32 | 24 | 9 | **108** |  |
| DRB1*0102 | 22 | 20 | 25 | 24 | 12 | **103** |  |
| DRB1*0301 | 24 | 27 | 20 | 21 | 32 | **124** |  |
| DRB1*0401 | 20 | 35 | 33 | 16 | 28 | **132** |  |
| DRB1*0403 | 21 | 27 | 21 | 22 | 45 | **136** |  |
| DRB1*0407 | 29 | 21 | 18 | 20 | 58 | **146** |  |
| DRB1*0701 | 31 | 30 | 30 | 31 | 26 | **148** |  |
| DRB1*0802 | 32 | 44 | 25 | 25 | 16 | **142** |  |
| DRB1*0901 | 32 | 29 | 26 | 28 | 57 | **172** |  |
| DRB1*1101 | 29 | 31 | 25 | 27 | 9 | **121** |  |
| DRB1*1104 | 38 | 22 | 33 | 17 | 8 | **118** |  |
| DRB1*1301 | 19 | 31 | 17 | 23 | 22 | **112** |  |
| DRB1*1402 | 18 | 25 | 4 | 22 | 53 | **122** |  |
| DRB1*1406 | 25 | 16 | 7 | 23 | 56 | **127** |  |
| DRB1*1501 | 29 | 24 | 22 | 25 | 20 | **120** |  |
| **Total peptides per serotype** | **395** | **399** | **338** | **348** | **451** | **1931** |  |

**Supplemental table 2. Statistical comparison of DENV CD4 MP180 reactivity among the different cohort analyzed in this study.** In blue are shown p values obtained by the comparison of the frequency of responders by Fisher’s test. In pink are shown p values for comparison of the magnitude of the responses by Mann-Whitney U test.

|  |  | **Frequency** | | | | |
| --- | --- | --- | --- | --- | --- | --- |
|  |  | **Singapore** | **India** | **Brazil** | **Nicaragua** | **Sri Lanka** |
| **Magnitude** | **Singapore** |  | **0.03** | 1.00 | 0.31 | 0.09 |
|  | **India** | **0.02** |  | **0.03** | 0.31 | 0.73 |
|  | **Brazil** | **0.01** | **0.002** |  | 0.18 | **0.05** |
|  | **Nicaragua** | 0.98 | 0.06 | **0.02** |  | 0.75 |
|  | **Sri Lanka** | 0.40 | 0.30 | **0.002** | 0.40 |  |


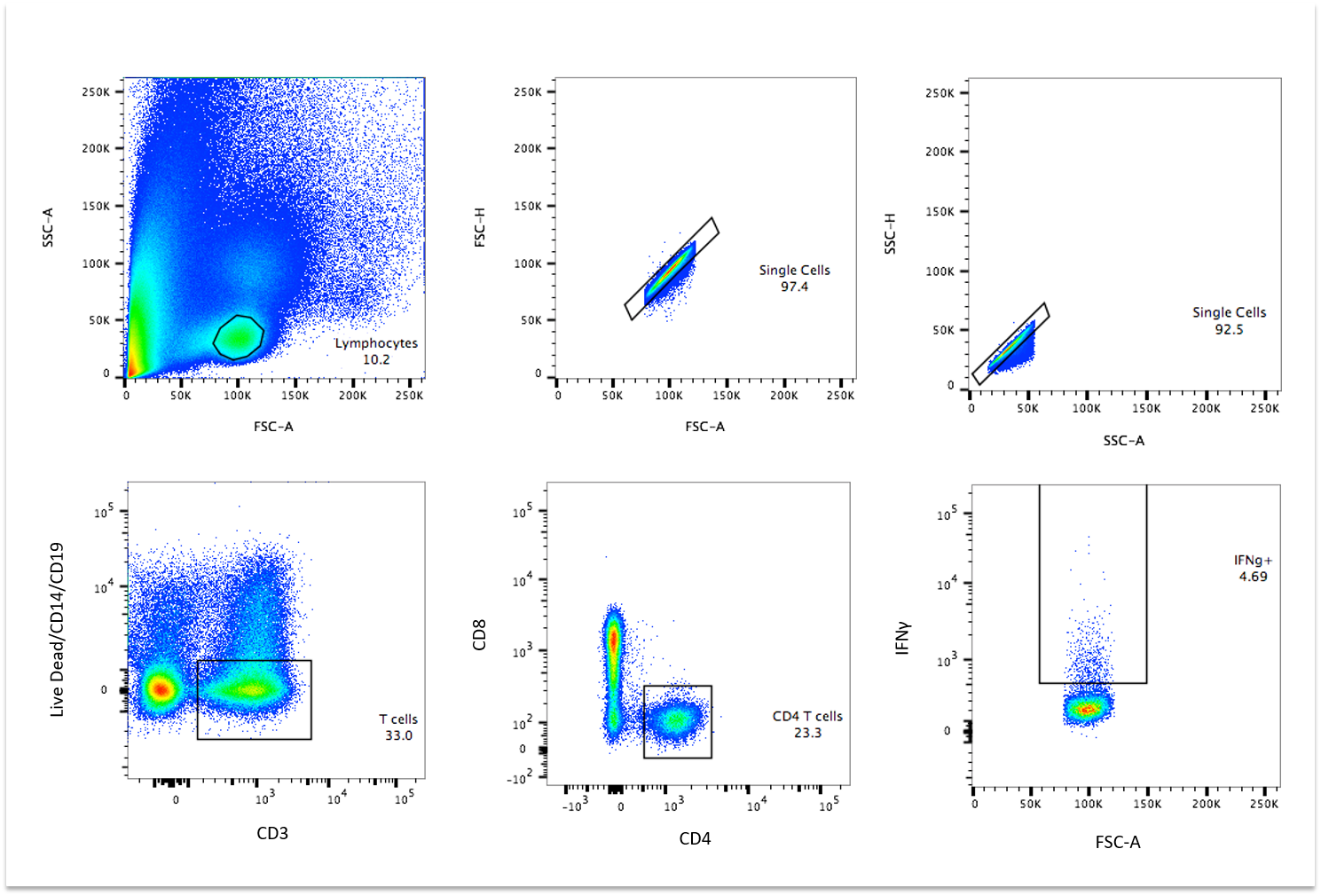


Figure S1. Gating strategy for ICS experiments.


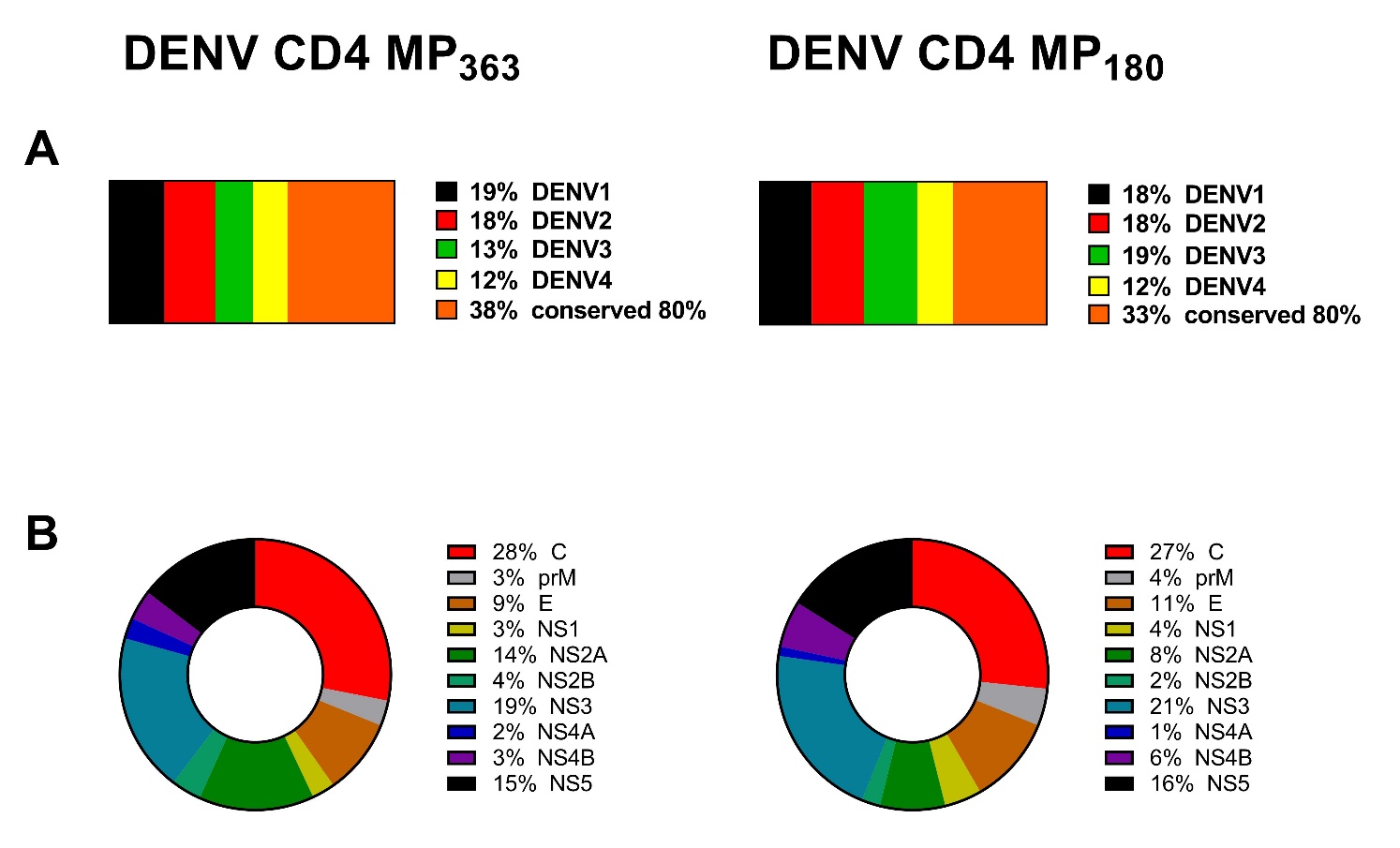


**Figure S2. Comparison of the two DENV CD4 MPs.** A) Serotype coverage. B) Protein composition.
